# The impact of shared decision-making on the treatment of anxiety and depressive disorders: systematic review

**DOI:** 10.1192/bjo.2021.1028

**Published:** 2021-10-07

**Authors:** Tyler Marshall, Chelsea Stellick, Adam Abba-Aji, Richard Lewanczuk, Xin-Min Li, Karin Olson, Sunita Vohra

**Affiliations:** Department of Psychiatry, Faculty of Medicine & Dentistry, University of Alberta, Canada; Department of Psychiatry, Faculty of Medicine & Dentistry, University of Alberta, Canada; Department of Psychiatry, Faculty of Medicine & Dentistry, University of Alberta, Canada; Department of Medicine, Faculty of Medicine & Dentistry, University of Alberta, Canada; and Department of Primary Health Care, Alberta Health Services, Canada; Department of Psychiatry, Faculty of Medicine & Dentistry, University of Alberta, Canada; Faculty of Nursing, University of Alberta, Canada; Department of Psychiatry, Faculty of Medicine & Dentistry, University of Alberta, Canada; and Department of Pediatrics, Faculty of Medicine & Dentistry, University of Alberta, Canada

**Keywords:** Clinical governance, ethics, comorbidity, anxiety disorders, depressive disorders

## Abstract

**Background:**

Shared decision-making encourages patients to explore treatment options/choices in collaboration with their healthcare provider, inclusive of the best available evidence and the patient's values/preferences. Several effective treatments exist for people with anxiety and/or depressive disorders; shared decision-making may be particularly useful in this context.

**Aims:**

To investigate whether shared decision-making enhances clinical outcomes in adults with anxiety and/or depressive disorders.

**Method:**

A systematic review was conducted. Five electronic health databases were searched from database inception until August 2019, in addition to reference lists of included studies. Prospective controlled studies of shared decision-making in adults (aged 18–64 years) diagnosed with an anxiety and/or depressive disorder were included. Two reviewers independently conducted each stage of the review process.

**Results:**

Six randomised controlled trials (*N* = 1834 participants) were included. Patient satisfaction improved in four studies. Patients were more likely to receive adequate treatment for depression in three studies. Anxiety symptoms decreased in one study. Patient involvement in decision-making increased in three studies. Because of the lack of blinded interventions and outcome assessment, the included studies were at moderate risk of bias. The certainty of evidence ranged from low to moderate, per GRADE criteria.

**Conclusions:**

Shared decision-making shows promise for enhancing quality-of-care outcomes such as patient satisfaction, without increasing consultation time. but appears unlikely to improve symptoms of depression. However, it appears to be understudied in patients with anxiety disorders. Heterogeneity regarding definition and measurement of shared decision-making posed challenges for interpreting the results. More research is recommended to advance the field.

## Background

Shared decision-making (SDM) has been described by Elwyn et al as ‘an approach where clinicians and patients share the best available evidence when faced with the task of making decisions, and where patients are supported to consider options to achieve informed preferences’.^1^ SDM has been recommended by the World Health Organization for reducing the global burden of chronic diseases,^2^ and is commonly considered to be the foundation of person-centred care.^3,4^

Previous research suggests that SDM may be associated with enhanced patient-reported outcomes, such as satisfaction with care, patient activation, patient knowledge gain, self-care and health-related quality of life, particularly among adults with chronic diseases such as diabetes, cardiovascular disease and dementia.^5–7^ Some scholars have advocated for the use of SDM in patients with mental health disorders;^8,9^ however, evidence for SDM in patients with mental health conditions has been less convincing. For example, in 2018, Samalin et al^10^ systematically reviewed the impact of SDM on various mood disorders, such as dysthymia, major depressive disorder and bipolar disorder. Fourteen randomised controlled trials (RCTs) were included, but only one study suggested that SDM, when facilitated by decision aids, may improve depressive symptoms, patient knowledge and health-related quality of life. The authors found only three studies investigating SDM among adults with depressive disorders, and studies of SDM in patients with anxiety disorders were excluded.^10^ In 2020, Fisher et al^11^ conducted a systematic review evaluating the effect of SDM among patients with mental ill health and substance/alcohol use disorder comorbidities. The authors found ten studies cautiously suggesting that SDM may be ‘acceptable, feasible and beneficial’ in this population.^11^ A recent meta-analysis suggests that ‘patient-centred communication’ may increase therapeutic alliance.^12^

## Rationale for review

As a result, we believe that is worthwhile to explore whether SDM may be beneficial for individuals with anxiety and/or depressive disorders. SDM may be suitable in this population for various reasons. For instance, anxiety and/or depressive disorders commonly co-occur, and are often treated by using a combination of various pharmacotherapeutic and psychotherapeutic approraches.^13,14^ Several classes of antidepressants exist, with each showing comparable safety and effectiveness.^15–18^ Additionally, several types of effective psychotherapies exist, such as cognitive–behavioural therapy, interpersonal therapy, dialectical behaviour therapy, behavioural activation therapy and problem-solving therapy, among others.^19^ Therefore, involving the patient in decisions pertaining to treatment selection may be largely based on the patient's personal preferences, and is unlikely to have a deleterious effect on health outcomes when the treatment regime is evidence-based.^20^

## Aims

Here, we aimed to investigate whether SDM enhances clinically relevant outcomes in adults (aged 18–64 years) with anxiety and/or depressive disorders. This systematic review provides a contribution to the literature by (a) using a novel, evidence-based conceptualisation and operationalisation of SDM; and (b) assessing clinically relevant outcomes identified by an expert panel of clinicians.

## Method

The protocol for this review is published in the PROSPERO International Prospective Register of Systematic Reviews (https://www.crd.york.ac.uk/PROSPERO), under registration number 126079. The Preferred Reporting Items for Systematic Reviews and Meta-Analyses (PRISMA) Statement^21^ was consulted to guide the conduct and reporting of this review. Since this was a secondary analysis of published data, ethics approval was not required.

### Eligibility criteria

Table 1 displays the full eligibility criteria. The *a priori* inclusion/exclusion criteria was adapted from the Population, Intervention, Control, Outcome, Setting (PICOS) model.^22^

**Table 1:** Inclusion/exclusion criteria

	Included	Excluded
Population	Adults aged ≥18 years Primary diagnosis of any anxiety or depressive disorder Cormoribd substance use disorder Comorbid physical health conditions (e.g. diabetes, cardiovascular disease) In-patients or out-patients	Studies with a mean age over 64 years Diagnosis of schizophrenia Diagnosis of bipolar disorder Patients mandated to treatment Patients at risk of harm to self or others (e.g. suicidal ideation)
Intervention	SDM according to the Elwyn et al^25^ criteria	Studies where the use of SDM was not clear
Control	Any	Studies where SDM was present in both treatment and control arms Uncontrolled studies
Outcomes	Any clinically relevant patient outcome	Healthcare provider outcomes
Study type	Randomised or non-randomised prospective controlled trials	Systematic reviews Observational studies Qualitative studies Case reports Expert opinion articles Grey literature

SDM, shared decision-making.

#### Participants

Studies of adults (aged >17 years) diagnosed with either an anxiety and/or depressive disorder were included. Studies of participants aged <18 years were excluded because a proxy decision maker is often required.^23,24^ Studies of older adults (aged >64 years) were excluded as this population was outside the scope of the review. Studies of patients with other mental and/or physical health comorbidities were included, except for severe mental illnesses such as bipolar disorder, schizophrenia or suicidality. Patients mandated to treatment, for any reason, were excluded.

#### Intervention

The intervention must have an equivalent to SDM as per the definition from Elwyn et al.^25^ We used the following criteria to screen eligible studies: patients were provided options or choices regarding their healthcare; patients and healthcare providers made decisions together, informed by the best available evidence; and patients’ preferences were considered by the healthcare provider.

In cases of uncertainty, we contacted the corresponding author of the study to obtain confirmation that the intervention met our *a priori* criteria for SDM.

#### Control

Only studies with a control group (e.g. active controls, sham controls, treatment-as-usual) were included.

#### Outcomes

Studies must have reported patient outcomes for eligibility. For example, studies that only reported healthcare provider outcomes were excluded.

#### Study type

Only prospective controlled trials published in peer-reviewed journals were included.

### Search strategy

A health research librarian assisted with developing and conducting the search strategy. Medline, EMBASE, PsycINFO, the Cochrane Database for Controlled Trials and the Cochrane Database for Systematic Reviews were searched from database inception until 18 August 2019. Reference lists of included texts were searched to ensure any remaining relevant articles were identified. Because of feasibility restraints, only studies published in English were considered. The search strategy used free-text and medical subject headings derived from a relevant scoping review.^26^ Supplementary File 1 available at https://doi.org/10.1192/bjo.2021.1028 displays the preliminary Medline search strategy.

### Study selection

Endnote for Windows (Version X9, Clarivate Analytics, Philadelphia, PA, US; see http://www.endnote.com) was used to manage the references and full-text PDFs. One reviewer screened the titles and abstracts, and another reviewer screened the excluded articles to ensure relevant articles were not inadvertently discarded. Two reviewers then independently reviewed the full text of each article to assess the inclusion/exclusion criteria. A third reviewer was consulted to arbitrate any disagreements.

### Data extraction

The included references were exported into a Microsoft Excel spreadsheet to complete the data extraction process. The data extraction form was developed *a priori* and piloted using two randomly selected studies. The data extraction form was finalized on 18 November 2019. Two reviewers independently extracted data from the remaining included studies. We contacted the corresponding authors of the included studies to request any missing data. Any discrepancies in data extraction between the reviewers were resolved by consensus.

### Data items

The following data items were extracted: bibliographic information (first author, title, year of publication and country); general study characteristics (study objectives, study design, setting, duration, data collection information); participant characteristics (number of patients, number of healthcare providers, age range, mean age, gender, diagnosis); methodological characteristics (number patients allocated to intervention and control, description of intervention, description of control, description of SDM measurement, reported outcomes); and main findings (effect size, *P*-value, drop-out rate, adverse events, author's conclusion and limitations).

### Risk of bias within studies

Two authors independently assessed the risk of bias for each study, using the Cochrane Risk of Bias Tool.^27^ Disagreements were resolved by discussion. The results of the risk-of-bias assessment were produced in Review Manager (RevMan) for Windows (Version 5.4. The Cochrane Collaboration, Copenhagen, DEN; see http://www.training.cochrane.org). The quality of the outcome evidence was assessed by two independent reviewers, using the Grading of Recommendations Assessment, Development, and Evaluation (GRADE)^28^ tool. A third reviewer was available to arbitrate any disagreements.

### Risk of bias across studies

An assessment of publication bias was planned with a funnel plot, where feasible.

### Synthesis of results

A meta-analysis was planned if a sufficient number of clinically homogenous articles were retrieved. Alternatively, a narrative synthesis was planned if meta-analysis was not possible.

### Additional analyses

Three subgroup analyses were conducted. First, a subgroup analysis of studies of emerging adults (individuals aged 18–25 years) was conducted *a priori.* Corresponding authors of the included studies were contacted to request data on this age group when possible. This age group was selected because there is reason to suspect that emerging adults may be more receptive to SDM compared with older adults.^29,30^ For example, emerging adults have shown to be more likely to prefer autonomy in health decision-making and self-management of mental health symptoms.^10,31^ Moreover, anxiety and depressive disorders are widespread globally, and are becoming increasingly common in this age group,^32^ but seldom receive timely and adequate care.^32,33^

Second, we conducted a *post hoc* analysis comparing studies of collaborative care involving SDM with studies with SDM interventions without collaborative care. Collaborative care is considered a patient-centred approach, involving the use of a multidisciplinary behavioural healthcare team, typically led by a primary healthcare provider.^34–36^ Collaborative care typically recommends incorporating patient goals into the treatment plan, but may not explicitly recommend or measure SDM.^37^

Third, studies of SDM facilitated by decision aids were analysed separately in a *post hoc* analysis. This analysis may be useful because it is unclear whether decision aids enhance the effects of SDM. Some research suggests that SDM does not require the use of a physical decision aid to be effective.^38^ Other studies have suggested that decision aids may promote SDM,^39^ and decision aids have been recommended by Elwyn et al^1^ to facilitate evidence-based decision-making when engaging in SDM.

## Results

### Study selection

**Fig. 1:**
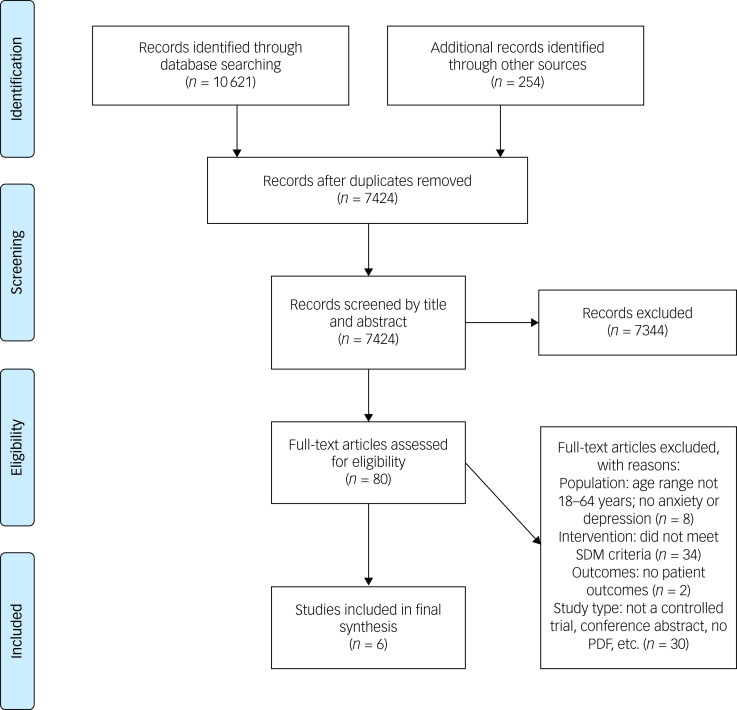
Preferred Reporting Items for Systematic Reviews and Meta-Analyses flow chart. SDM, shared decision-making.

Figure 1 illustrates the study selection process. The search of electronic health databases initially retrieved 10 621 publications. After discarding duplicates, we obtained 7424 references, which were screened by title and abstract. Eighty articles were retained for screening in full text. As part of this evaluation, we contacted the corresponding authors of 19 studies to verify whether the trialled intervention met our *a priori* definition for SDM. Three additional studies^40–42^ were included as a result. Supplementary File 2 displays the results of this inquiry. Six studies were included for the final data synthesis.

### Characteristics of included studies

Table 2 displays the characteristics of included studies. Six RCTs^40–45^ met the inclusion criteria (*N* = 1834 participants). The publication dates ranged from 2007 to 2015. All six studies included patients who were diagnosed with a depressive disorder; only two studies included patients^41,42^ with anxiety disorders. Four of the six studies^40–42,44^ were conducted in the USA, one in Germany^45^ and one in Saudi Arabia.^43^ Three of the six studies were parallel RCTs^41–43^ and three were cluster RCTs^40,44,45^ in which physician clinics, rather than participants, were randomised to the intervention group. Two studies were conducted in an in-patient hospital setting,^42,43^ three studies were conducted in primary care settings^40,44,45^ and one study was conducted at a public health centre.^41^ The patient population varied considerably. Three studies^40,44,45^ involved adults (aged 18–64 years) with depression in primary care. One study^41^ included only women with perinatal depression and low socioeconomic status. Another study^42^ included only in-patients with moderate-to-severe depression in addition to comorbid cardiovascular disease.

**Table 2:** Characteristics of included studies

Study, year (country)	Study design	Inclusion criteria	Sample size	Duration of study	Setting	Age range, mean age (s.d.), % male	Description of intervention (number of participants)	Description of control conditions (number of participants)	Outcomes	Outcome measurement instruments	Data collection intervals
Aljumah et al, 2015^43^ (Saudi Arabia)	Parallel RCT	Major depressive disorder (DSM-IV), no history of psychosis or bipolar disorders, no drug or dependency history, no cognitive impairment	*n* = 239 patients	11 months	In-patient	18–60 years, mean not reported, 41% male	Shared decision-making facilitated by pharmacist intervention focused on enhancing patients’ involvement in decision-making by assessing their beliefs and knowledge about antidepressants (*n* = 119)	Usual pharmacy services (*n* = 120)	-Medication adherence -Patient's beliefs about medicine -Depression severity -Patient involvement in decision-making -Health-related quality of life -Patient satisfaction with treatment	-MMAS (mean scores) -BMQ (specific and general versions, mean scores) -MADRS (mean scores) -OPTION (overall scores*) -EuroQol-5D (mean scores) -TSQM 1.4 (mean scores)	Baseline, 3-month follow-up, 6-month follow-up
Chaney et al, 2011^40^ (USA)	Cluster RCT	Major depressive disorder (PHQ-9 ≥ 10), no acute suicidality	*n* = 546 patients	21 months	Primary care	Mean age 64.0 years intervention, mean age 64.4 years control, 95.8% male intervention, 96.5% male control	EBQI applied to collaborative care model implementation (*n* = 288)	Usual care: non-collaborative care model (*n* = 258)	-Receipt of appropriate treatment -Depression severity -Patients below threshold for major depression -Physical functional status -Emotional functional status -Satisfaction with mental healthcare	Antidepressant fill at appropriate dosage in the 7-month time period, and the medication possession ratio -PHQ-9 (mean scores) -PHQ-9 (percentage with a score of <10) -SF-12 (mean role physical scores) -SF-12 (mean role emotional scores) -Percentage that are somewhat or very satisfied	Baseline, 7-month follow-up
Grote et al, 2015^41^(USA)	Parallel RCT	Aged ≥18 years, diagnosis of probable major depressive disorder or probable dysthymia	*n* = 168 patients	49 months	Public health centres	18–44 years, mean 27.4 (6.1) years, 0% male	MOMCare, a collaborative care intervention, providing a choice of brief interpersonal psychotherapy and/or antidepressant therapy (*n* = 83)	Usual care: Maternity Support Services (*n* = 85)	-Depression severity -Functional impairment -Treatment response -Complete remission of depressive symptoms -PSTD severity -Probability of GAD diagnosis -Quality of mental healthcare (number of sessions, antidepressant use, satisfaction with care)	-SCL-20 (mean scores) -WSAS (main effects) -SCL-20 (≥50% reduction from baseline score) -SCL-20 (score <0.5) PCL-C (main effects)-PHQ (overall score) -standardised questions and antidepressive use	Baseline, 3-, 6-, 12- and 18-month follow-up assessments
Huffman et al, 2011^42^(USA)	Parallel RCT	Acute cardiac disease, depression (PHQ-9 ≥ 10) and five or more symptoms including depressed mood or anhedonia	*n* = 175 patients	28 months	In-patients	Mean 62.3 (12.5) years, 51.4% male	Collaborative care: the care manager performed a multicomponent depression intervention in the hospital that included patient education and treatment coordination (*n* = 90)	Usual care: in-patient providers were informed of the depression diagnosis (*n* = 85)	-Adequate depression treatment by discharge	Discharge prescription of an antidepressant at a clinically effective dose based on manufacturers’ package labeling and treatment guidelines for the treatment of depression, or referral to a mental health treatment provider for psychotherapy	Baseline, 6-month follow-up
LeBlanc et al, 2015^44^(USA)	Cluster RCT	Moderate-to-severe depression and a PHQ-9 score of 10 or higher, no bipolar disorder, an appointment with a member of their primary care team and no major barriers to providing informed consent	*n* = 301 patients, *n* = 30 clinicians	24 months	Primary care	>18 years, mean 44 (15) years, 33% male	Shared decision-making facilitated by physician use of a decision aid during selection of antidepressants (*n* = 158)	Usual care (*n* = 139)	-Decisional conflict -Patient knowledge and satisfaction -Patient involvement in decision-making -Depression (symptoms, remission, responsiveness) -Primary medication adherence -Secondary medication adherence -Encounter duration -Satisfaction	-DCS (overall mean scores) -Post-encounter questionnaire -OPTION (scoring recorded encounters) -PHQ-9 (mean scores, scores <5, >50% improvement) -Percent that filled prescription -Proportion of days covered >80% -Mean duration in minutes -Investigator developed five-point Likert scale	Baseline, 3-monthand 6-month follow-up
Loh et al, 2007^45^(Germany)	Cluster RCT	Diagnosis of depression (PHQ-9), no psychotic symptoms, functional language and literacy abilities	*n* = 405 patients, *n* = 117 clinicians	27 months	Primary care	Mean 40.8 (13.2) to 50.4 (16.3) years, 22.2–34.7% male	Shared decision-making facilitated by used of a decision aid during selection of antidepressants (*n* = 263)	Usual care (*n* = 142)	-Patient involvement -Depression severity and clinical outcome -Treatment adherence -Patient satisfaction -Consultation time	-PICS and MSH scale (pre–post intervention) -Brief PHQ-D (percentage reduction in severity) -Investigator developed five-point Likert scale (patient and physician) -CSQ-8 -Duration in minutes (documented by physician)	-Baseline, 6-8 week follow-up

RCT, randomised controlled trial; MMAS, Morisky Medication Adherence Scale; BMQ, Patients’ Beliefs about Medicine Questionnaire (specific and general versions); MADRS, Montgomery–Åsberg Depression Rating Scale; OPTION, Observing Patient Involvement in Decision-Making Scale; TSQM 1.4, Treatment Satisfaction Questionnaire for Medication; PHQ, Patient Health Questionnaire; EBQI, Evidence-Based Quality Improvement; SF-12, Medical Outcomes Study Short Form; PTSD, post-traumatic stress disorder; GAD, Generalized Anxiety Disorder; SCL-20, Hopkins Symptom Checklist; WSAS, Work and Social Adjustment Scale; PCL-C, Post-Traumatic Stress Disorder Checklist-Civilian Version; DCS, Decisional Conflict Scale; PICS, Patient's Perceived Involvement in Care Scale; MSH, Man-Son-Hing instrument; PHQ-D, Patient Health Questionnaire-Depression; CSQ-8, Client Satisfaction Questionnaire.

Three of the six studies^43–45^ employed interventions where SDM was explicitly defined *a priori*. Two of these^43,44^ quantitatively measured the SDM with a validated instrument such as the Observing Patient Involvement in Decision-Making Scale (OPTION) tool,^46^ and one study^45^ measured SDM by using a combination of the Patient Perceived Involvement in Care Scale^47^ and the patient participation scale (Man-Son-Hing instrument). One study^43^ assessed a pharmacist intervention based on SDM that provided direct patient care compared with usual pharmacy services. The other two studies that explicitly defined SDM^44,45^ involved the use of an evidence-based decision aid for antidepressants, compared with primary care as usual with no decision aid. Three studies^40–42^ consisted of collaborative care interventions in which SDM was confirmed to be incorporated into the intervention by the corresponding authors. Collaborative care was generally delivered as a multicomponent depression intervention, including patient education and treatment coordination led by a patient care manager.^40-42^

### Risk of bias within studies

**Fig. 2:**
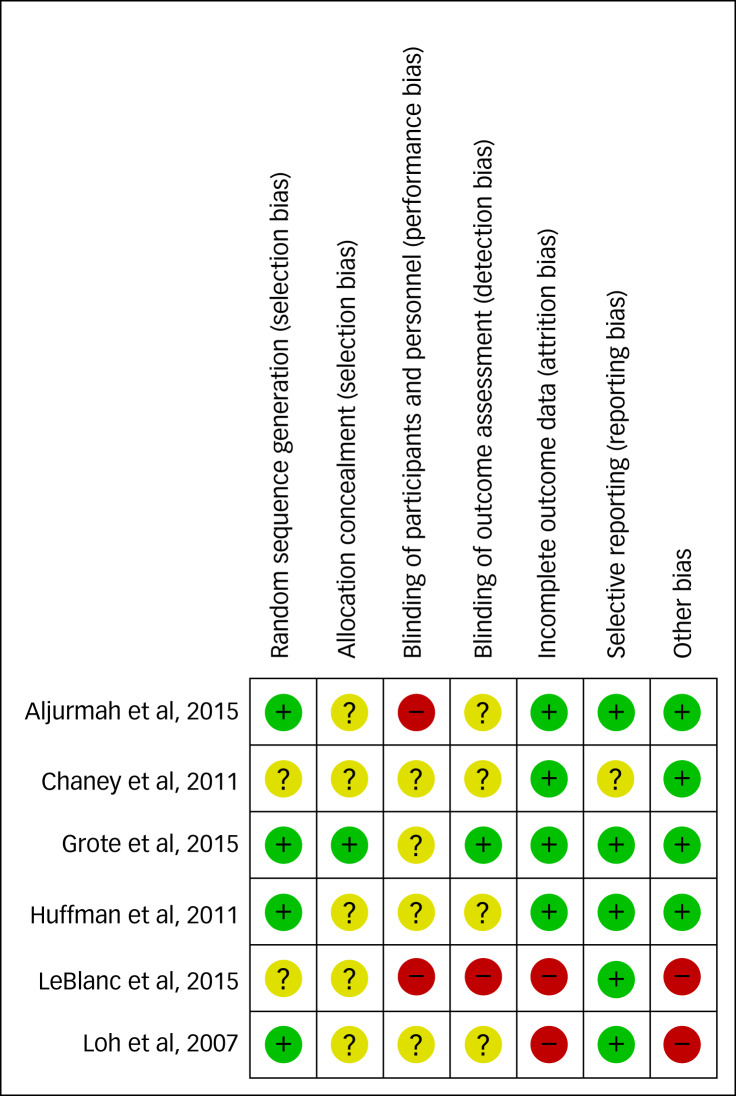
Risk-of-bias (ROB) summary of results. Legend: green, low ROB; red, high ROB; yellow, unclear ROB.^40–45^

Figure 2 displays the results of the risk-of-bias assessment. The included RCTs were at moderate risk of bias. Two studies did not report random sequence generation methods;^40,44^ five studies did not report allocation concealment strategies;^11,40,42–45^ two studies^43,44^ did not blind the participants or outcome assessors, and three studies did not provide a clear description;^40,41,45^ one study^44^ did not adequately blind the outcome assessor, three studies were unclear;^40,42,45^ two studies^44,45^ suffered from incomplete reporting of outcome data, and one study did not clearly pre-specify outcomes.^40^ No other sources of bias were identified.

### Risk of bias across studies

Assessment of publication bias was not conducted because of an insufficient number of included studies (fewer than ten) per Cochrane recommendations.^48^

### Impact of SDM on clinically relevant outcomes

Table 3 displays the summary of findings according to patient outcome.

**Table 3:** Summary of main findings according to patient outcome

	Anxiety symptoms	Depressive symptoms	Quality of life	Treatment adherence	Adequate depression treatment	Patient knowledge	Patient satisfaction	Involvement in decision-making
Number of reporting studies	1	5	1	3	3	1	5	3
Increased (*P* < 0.05)	0	0	0	1	3	1	4	3
Decreased (*P* < 0.05)	1	1	0	0	0	0	0	0
No change (not significant)	0	4	1	2	0	0	1	0
Quality of evidence, GRADE	Low	Moderate	Low	Low	Moderate	Low	Moderate	Moderate
Reasons for downgrade	Indirectness Imprecision	Inconsistency	Imprecision	Inconsistency	Risk of bias	Imprecision	Inconsistency	Imprecision

GRADE, Grading of Recommendations Assessment, Development, and Evaluation.

#### Symptoms of anxiety

Only one of the six included studies^41^ reported whether SDM affected symptoms of anxiety. Grote et al^41^ found that a collaborative care intervention inclusive of SDM (MOMCare and Maternity Support Services-Plus (MSS-Plus), *n* = 83 participants) reduced the number of participants who met the criteria for anxiety on the Generalized Anxiety Disorder-7 questionnaire compared with the control condition (MSS-Plus, *n* = 81 participants) at the 18-month follow-up (*n* = 152; intervention 10.0% *v*. control 22.2%; odds ratio 0.39; 95% CI 0.16−0.97). Although there was a medium effect size of 0.52, only one study reported this outcome, resulting in low certainty of evidence owing to imprecision in using the GRADE criteria.^49^

#### Symptoms of depression

Five of the six included studies^40,41,43–45^ reported whether SDM affected symptoms of depression. Grote et al^41^ reported that a collaborative care intervention inclusive of SDM (MOMCare) was associated with a decrease in mean depression severity on the 20-item Hopkins Symptom Checklist (SCL-20) compared with the control (MSS-Plus) at the 6-month follow-up (*n* = 157; mean difference −0.24; 95% CI −0.46 to 0.03; *P* = 0.03) and 18-month follow-up (*n* = 152; mean difference −0.25; 95% CI −0.45 to 0.04; *P* = 0.02). Four studies^40,43–45^ reported no change in depression severity compared with control conditions. Depression was measured in a variety of ways, including depression severity, using the Patient Health Questionnaire-9, SCL-20 and Hospital Anxiety and Depression Scale. Given the variation in outcome results, the certainty of the evidence was rated moderate because of inconsistency.^49^

#### Health-related quality of life

One of the six studies^42,43^ reported whether or not SDM affected health-related quality of life. Aljumah et al^43^ reported no change in health-related quality of life resulting from usual pharmacy services plus pharmacist interventions based on SDM compared with usual pharmacy services after 6 months of follow-up. Health-related quality of life was measured with the EuroQol-5D. Given that only one included study was available for the GRADE rating and the study results were inconclusive, the certainty of the evidence on health-related quality-of-life outcomes was rated low because of imprecision.^37^

#### Likelihood of receiving evidence-based depression treatment

Three research teams^40–42^ reported whether SDM affected the probability of receiving adequate depression treatment. Adequate depression treatment was defined by Huffman et al^42^ as either prescription of an antidepressant at a clinically effective dose according to treatment guidelines, or referral to a mental health treatment provider for psychological therapy. This was measured by the medication possession ratio^50^ and by obtaining information from patient charts.

The researchers^40–42^ also found that the collaborative care models involving SDM increased the likelihood of receiving either antidepressant therapy or referral to psychological therapy. Chaney et al^40^ found that the evidence-based quality improvement collaborative care model (EBQI-CCM) intervention (*n* = 268) improved the likelihood of receiving an adequate dosage of antidepressant therapy (65.7% received adequate dosage) at 7 months after baseline compared with the non-EBQI-CCM intervention (*n* = 238, 43.4% received adequate dosage; difference 22.3, *P* < 0.001). Grote et al^41^ reported that the MOMCare and MSS-Plus (*n* = 83) intervention group had higher rates of antidepressant use regarding group (*χ*² = 8.10, d.f. = 1, *P* < 0.01) and time (*χ*² = 18.67, d.f. = 3, *P* < 0.0001) effects, compared with the MSS-Plus control group (*n* = 81). The intervention group also displayed a higher adherence rate than the control group, with statistical significance (*χ*² = 10.00, d.f. = 1, *P* = 0.002). In addition, Huffman et al^42^ found that the collaborative care intervention group (*n* = 90) had a significantly higher likelihood of being prescribed adequate depression treatment compared with the usual care control group (*n* = 85) (intervention: 64 out of 89 (71.9%) *v*. control: 8 out of 84 (9.5%); *χ*² = 71.46; d.f. = 1; *P* < 0.001). The effect size was 0.66 at 3 months, 0.52 at 6 months, 0.54 at 12 months and 0.05 at 18 months. The certainty of the evidence was downgraded to moderate because of risk of bias among the included studies.

#### Patient satisfaction with care

The research teams of five of the six studies^40,41,43–45^ discussed whether SDM affected patient satisfaction with care. Four groups^41,43–45^ found an increase in patient satisfaction as a result of SDM interventions, whereas one group^40^ found no statistically significant difference. Aljumah et al^43^ indicated that after 3 months of treatment, the group that received pharmacy services based on SDM (*n* = 110) had significantly higher scores on treatment satisfaction than the group that received usual pharmacy services (*n* = 110; *t* = 2.326, *P* = 0.021). After 6 months of treatment, the effect on treatment satisfaction was still statistically significant (*t* = 3.551, *P* < 0.0001). Grote et al^41^ found that the MOMCare group participants (*n* = 83) reported higher mean levels of satisfaction compared with the MSS-Plus group (*n* = 81) at follow-up, with no significance in the main effect for time (*χ*² = 0.36, d.f. = 3, *P* = 0.95), but statistical significance for group main effect (*χ*² = 8.28, d.f. = 1, *P* = 0.004).

LeBlanc et al^44^ reported patients in the decision aid group (*n* = 158) expressed higher overall satisfaction with treatment compared with the control group (*n* = 139) (relative risk ranging from 1.25 (*P* = 0.81) to 2.40 (*P* = 0.002)). There was no significant effect found between groups on the whether the ‘right amount of information was given’ (*P* = 0.81) or ‘information given was extremely clear’ (*P* = 0.09). Loh et al^45^ found that patients in the patient-centred decision aid intervention group (*n* = 128) had significantly higher satisfaction levels at the post-intervention stage compared with the control group (*n* = 66; *P* = 0.014). However, since this questionnaire measurement tool was not administered at baseline, no temporal comparison of differences in satisfaction was possible. Chaney et al^40^ found no statistical significance for overall patient satisfaction; the author stated that 62.4% of the EBQI-CCM intervention group (*n* = 288) reported being ‘satisfied or very satisfied with mental healthcare’. Moreover, 67.3% of the non EBQI-CCM group (*n* = 258) answered the survey question affirmatively (*P* = 0.27).

#### Treatment adherence

Three of the six research teams^43–45^ studied the influence of SDM on treatment adherence. In one case,^43^ the intervention was a provided by a pharmacist and reported a significant difference between the intervention and the control group. The authors of the other two studies reported no significant difference between treatment groups. Aljumah et al^43^ found that at the 3-month follow-up, the intervention group (*n* = 110) reported significantly higher scores for medication adherence compared with the control group (*n* = 110; *t* = 2.88, *P* = 0.004). Statistically significant results were also observed at the 6-month follow-up (*t* = 4.0598, *P* < 0.001). LeBlanc et al^44^ reported that no clinical variation was found between treatment adherence percentages for patients in the decision aid arm (*n* = 158, 86.2%) and the control arm (*n* = 135, 93.2%) (*P* = 0.19). Loh et al^45^ found similar results, where no statistical differences in patient-reported adherence were found between the patient-centred intervention group (*n* = 191) and the control condition participants (*n* = 96; *P* = 0.73). This was also the case for physician-rated treatment adherence in the intervention and control groups (*P* = 0.56). Because of the inconsistency in outcome definitions and measurements across the articles, the certainty of the evidence was low.

#### Patient involvement in health decision-making

Three of the six research studies^41,44,45^ examined whether SDM affected patient involvement or engagement in health decision-making. Grote et al^41^ found that 97.5% of the participants in MOMCare intervention (*n* = 83) and MSS-Plus group (SDM) were engaged in treatment compared with 35.2% of the MSS-Plus group (*n* = 81; odds ratio 72.7, 95% CI 16.5−321). LeBlanc et al^44^ used cluster-adjusted *t*-tests and found that the decision aid group (*n* = 158) was significantly more involved in the decision-making, with 47% of the intervention group participants reporting involvement compared with 33% of the control group (*n* = 139; *P* = 0.001). The certainty of the evidence was low because of the inconsistency in the outcome measurement and reporting, and low number of included studies.

#### Consultation time

Two of the six research teams^44,45^ studied whether SDM affected consultation time. Both studies reported no change in consultation time because of using SDM facilitated by decision aids. LeBlanc et al^44^ found no clinically significant differences in clinical encounter duration between the decision aid intervention group (*n* = 158) and the control group (*n* = 139). The mean time was 44 (s.d. = 22) minutes for the intervention group, and 48 (s.d. = 27) minutes for the control group (*P* = 0.47). In addition, Loh et al^45^ reported that there was no statistical difference in clinical consultation time between the intervention (*n* = 191) and control groups (*n* = 96) for both within-group pre- and post-treatment comparisons (intervention: *P* = 0.48; control: *P* = 0.64), and between the treatment arms (*P* = 0.68). The quality of the evidence obtained was low because of inconsistency in reporting outcome data, and low number of included studies.

### Additional analyses

#### Emerging adults

No studies or data were obtained involving emerging adults.

#### SDM versus SDM and collaborative care

Three RCTs^43–45^ conducted investigations of SDM interventions without the use of collaborative care, and three RCTs^40–42^ conducted investigations of collaborative care interventions involving SDM. The studies involving collaborative care differed by using multiple healthcare providers, coordinated by a care manager to evaluate and coordinate the personalised care of individuals with depression based upon unique needs.^40–42^ Studies of SDM alone^43–45^ primarily involved interactions between one healthcare provider and the patient.

All three RCTs^40–42^ using collaborative care models found that patients with depression were more likely to receive adequate depression treatment compared with usual care without SDM, with statistical significance (Chaney et al^40^: *P* = 0.001; Grote et al^41^: *P* = 0.01; Huffman et al^42^: *P* < 0.001); however, the studies of SDM without collaborative care did not report this outcome. All three research groups who studied SDM^43–45^ found that SDM improved satisfaction with care, and one of the two groups who studied collaborative care^41^ reported that SDM improved satisfaction with care. Comparisons of other clinically relevant outcomes could not be conducted because of inconsistency in reporting.

#### SDM facilitated with a decision aid

Two research teams used decision aids to facilitate SDM compared with treatment as usual.^44,45^ No change was found in depressive symptoms or treatment adherence in either study. One research team who studied decision aids reported an increase in patient knowledge;^44^ both research groups who studied SDM and decision aids reported improvements in satisfaction with care. However, two of the three teams who studied SDM without decision aids^41,43^ also reported improved satisfaction with care. Both research teams who studied SDM with decision aids found that patient participation improved, but the only team studying SDM without decision aids^41^ also found that patient participation improved because of collaborative care. Both teams that studied SDM studies with decision aids reported no change in consultation time. Comparisons with studies without decision aids were not possible because of limitations in outcome reporting.

## Discussion

To our knowledge, this is the first systematic review to examine whether SDM affects clinically relevant outcomes in adults diagnosed with anxiety and/or depressive disorders. We found preliminary evidence suggesting that SDM may favourably affect outcomes such as satisfaction with care and patient involvement in decision-making. We found evidence that SDM, when used in conjunction with a collaborative care intervention, likely improves the likelihood of receiving adequate depression treatment, and preliminary evidence that SDM may facilitate a reduction in symptoms of anxiety. However, only one study reported anxiety as an outcome, and a statistically significant difference was only detected at 18 months of follow-up.^41^ It appears unlikely that SDM by itself improves symptoms of depression, as only one of the five studies that measured this outcome demonstrated a statistically significant improvement. The lone study^41^ that demonstrated an improvement also used collaborative care in addition to SDM, which may be partially responsible for the observed effect.

Among the included studies, only one study suggested that collaborative care involving SDM may improve symptoms of anxiety or depression.^41^ SDM and collaborative care studies appeared to show promising results on improving outcomes such as patient satisfaction with care, but the SDM and collaborative care studies largely measured different outcomes. Over the past 20 years, a considerable evidence base has grown in support of collaborative care for improving outcomes in patients with anxiety and depression. For example, several large systematic reviews and meta-analyses have shown that collaborative care is more effective than usual care in improving both quality-of-care outcomes and symptoms of anxiety and depression in both the short and long term.^35,36,51^ Therefore, we hypothesise that it is possible that collaborative care may either facilitate or enhance SDM.

Two included studies^25,52^ suggested that decision aids may facilitate SDM, but it was unclear from our data whether decision aids affected the effectiveness of SDM in our target population. We were only able to compare the effects of SDM facilitated via a decision aid on two outcomes (satisfaction with care and patient participation), and both approaches were beneficial. More research is needed to clarify the effect of decision aids on the effectiveness of SDM in people with anxiety and/or depressive disorders. Additionally, no relevant studies were found in the emerging adult population, and the corresponding authors of the included studies were not able to provide any additional data to conduct any further analysis. More research is needed to explore the potential effects of SDM in emerging adults, as there is evidence that emerging adults may avoid mental healthcare services and may benefit from approaches that facilitate increased engagement.^33,53–57^

The findings of our systematic review are largely consistent with recent systematic reviews of SDM in the treatment of other mental health disorders.^10,11,31^ Samalin et al^10^ found 14 RCTs that used SDM or collaborative care with SDM among patients with bipolar or depressive disorders. The authors suggested improvements in patient satisfaction and engagement in decision-making were consistent across studies. Fisher et al^11^ found ten studies of SDM in adults with co-occurring substance use and mental health disorders. This systematic review suggested that SDM was likely to be acceptable and feasible for patients with these conditions; more research was recommended by the authors.^11^ Neither systematic review specifically evaluated the effects of SDM in people with anxiety disorders or in emerging adults.

We aimed to expand upon this work by searching for SDM studies of anxiety disorders and conducting subgroup analyses around the effects of SDM in emerging adults, and by separately analysing studies aiming to facilitate SDM via a decision aid.^10^ In keeping with previous systematic reviews, we also found evidence that SDM primarily improved outcomes such as satisfaction with care and patient involvement in decision-making.^10,11,31^ Our review, however, highlights a substantial gap in the literature around SDM in mental healthcare, as only two studies^40,41^ included adults with anxiety disorders, and only one study^41^ measured symptoms of anxiety, which is insufficient for drawing firm conclusions. Future research in this area is warranted, as the study^41^ suggested that collaborative care intervention (with author-confirmed use of SDM) was associated with a decrease in anxiety and post-traumatic stress disorder symptom severity among socioeconomically disadvantaged women with perinatal depression.

As a result, we hypothesise that SDM may not directly ameliorate symptoms of anxiety or depression; rather, the effects of SDM may be mediated via improvements around patient-valued and/or -reported outcomes, such as therapeutic alliance, satisfaction with care, patient healthcare engagement and patient knowledge gain. Improvements in patient-reported outcomes may affect additional outcomes over time, such increased probability of selecting of appropriate treatments or medications, and adherence to these treatments, which in turn, may result in improved mental health outcomes.^18^

SDM may be particularly beneficial during deliberation and selection of various effective treatment options (e.g. pharmacotherapy and/or psychotherapy), or during selection of specific medications and/or psychotherapies.^24,44,48^ Therapeutic alliance and treatment adherence may improve when SDM is practiced consistently during each clinical encounter.^44^ A majority of included studies measured/assessed SDM during a single clinical encounter, thus precluding any ability to observe additional benefit SDM may provide when practiced longitudinally. As a result, we hypothesise that continued engagement with the patient in a person-centred approach over time may be more beneficial than a single engagement.^9,58,59^

Conversely, SDM may not be applicable in every clinical circumstance, particularly if there is an emergency situation and the patient is at risk of harm to self or others; the patient's illness significantly impairs decision-making capacity, clearly impeding the ability to make sound choices; or the patient prefers to defer decision-making to a healthcare provider.

In light of these circumstances, SDM may also be attempted with a proxy decision maker (e.g. family member or caregiver), if required. Future research is warranted to explore the effect of SDM when practiced frequently/consistently, clarify when SDM may be impossible/inappropriate, and identify ways to adapt/modify SDM during challenges clinical situations.

### Limitations of the included studies

The included studies were at moderate risk of bias, which weakened the certainty of the evidence. The primary issues were around concealment of allocation and inconsistently defined and measured SDM interventions. Moreover, to the best of our knowledge, there is no universally accepted definition of SDM in the literature. The lack of a consistently defined construct of SDM in the literature poses challenges for researchers to assess the occurrence and effectiveness of SDM across multiple studies.^60,40–42^ Additionally, the reporting of SDM remained unclear in several potentially eligible studies, posing challenges for the review. We recommend clear reporting of SDM and how it was measured in any future studies, regardless of whether SDM is the primary intervention.

Only two of the six studies measured SDM using a validated instrument such as the OPTION scale,^61^ therefore it was difficult to assess to what extent SDM was delivered, and whether or not SDM was present in the control groups. Although the three collaborative care studies involving SDM^40–42^ measured adequate depression treatment (i.e. prescribed an antidepressant based on clinical practice guidelines or referred to psychological therapy) as a primary outcome, none of the other SDM studies measured this outcome. Our observation is supported by a recent scoping review^62^ that also found inconsistencies among SDM outcomes and tools, which may ultimately reflect heterogeneity in how SDM is defined and measured. Validated and consistent outcome reporting in future clinical trials on SDM is needed to further synthesise the results required to better inform policy and clinical practice.

### Strengths and limitations of the review

Our study has several strengths. We performed a comprehensive search spanning five healthcare databases, using literature-informed search terms related to SDM, anxiety and depressive disorders. A unique aspect of this study was that we operationally defined SDM and created rigorous inclusion criteria based on this definition. Furthermore, we contacted authors of studies where it was unclear whether SDM occurred. Two independent reviewers performed all stages of screening and analysis. Additionally, this systematic review included several clinically relevant outcomes that may be useful for informing clinical practice and policy.

There are also several limitations of this review that may affect the interpretation of findings. SDM is sometimes studied in other study types such as observational or qualitative studies, and the exclusion of these study types may not provide a clear map of all evidence investigating SDM in adults with anxiety and depression. However, including studies with these methods would have increased methodological heterogeneity in the review, and would not have helped address our review objectives. Because of feasibility concerns, we did not search for literature outside of the English language, which may have limited the inclusion of potentially relevant studies. This limitation may weaken the generalisability of this review. Since the objective of this review was SDM, we did not design a search strategy around collaborative care. We acknowledge that collaborative care may sometimes involve SDM, and therefore these studies may have been missed in our search.

Clinical heterogeneity across the included studies posed challenges for data synthesis and the interpretation of the results. For example, three of the studies involved patients with depression from primary care settings, whereas the other three studies involved specific populations such as in-patients with cardiovascular disease, women with perinatal depression and psychiatric in-patients receiving care from pharmacists. Moreover, each of these studies delivered SDM in a unique way, which made making comparisons difficult and combining results via meta-analysis not possible. For example, three of the six studies^40–42^ used a collaborative care intervention in addition to SDM. Notably, the collaborative care approach in these studies involved multiple modalities in addition to SDM, which may have increased the likelihood of observing a significant effect. Furthermore, meta-analysis was also not possible because of inconsistency in outcome reporting and heterogeneity in both the measurement of the independent variable (e.g. SDM) and the outcome measurements. Additionally, because of the low number of included studies, we were not able to assess publication bias, which cannot be entirely dismissed.

Overall, the obtained evidence cautiously suggests SDM may have more benefits than risks in the treatment of adults with anxiety and/or depressive disorders. The low number of studies and high clinical heterogeneity among the included studies precludes drawing firm conclusions; however, our findings are consistent with previous systematic reviews on SDM in patients with other chronic conditions. More high-quality research that uses a consistent definition (and reliable measurements) of SDM is needed to advance knowledge in the field. Additional research around SDM in emerging adults with mental health concerns, and adults with anxiety disorders, is also recommended.

## Data Availability

The data that support the findings of this study are available from the corresponding author, S.V., upon reasonable request.

## Supplementary material

For supplementary material accompanying this paper visit http://dx.doi.org/10.1192/bjo.2021.1028. click here to view supplementary material
