# The impact of shared decision-making on the treatment of anxiety and depressive disorders: systematic review – CORRIGENDUM

**DOI:** 10.1192/bjo.2021.1050

**Published:** 2021-11-17

**Authors:** Tyler Marshall, Chelsea Stellick, Adam Abba-Aji, Richard Lewanczuk, Xin-Min Li, Karin Olson, Sunita Vohra

**Keywords:** Clinical governance, ethics, comorbidity, anxiety disorders, depressive disorders

In the original publication,^1^ a minor typographical error was made in the abstract. The first sentence of the conclusion in the abstract had a comma instead of a period as shown below “… without consultation time. but appears unlikely…”. This has now been corrected in both the online PDF and HTML versions of this article. The authors apologise for this error.
