# Supplementary material for: The impact of shared decision-making on the treatment of anxiety and depressive disorders: systematic review
Source: BJPsych Open. 2021 Oct 7;7(6):e189. doi: 10.1192/bjo.2021.1028 (PMC8517854; doi:10.1192/bjo.2021.1028)
Supplement: Supplementary file 1 [file bjosup.zip › S2056472421010280sup001.docx]

**S3.** **Deviations from protocol**

We also excluded studies of older individuals (mean age: 65+ years) as the treatment preferences and experience of anxiety and depressive disorders in older adults may not be comparable to younger populations.[63, 64]
