# Supplementary material for: The impact of shared decision-making on the treatment of anxiety and depressive disorders: systematic review
Source: BJPsych Open. 2021 Oct 7;7(6):e189. doi: 10.1192/bjo.2021.1028 (PMC8517854; doi:10.1192/bjo.2021.1028)
Supplement: Supplementary file 1 [file bjosup.zip › S2056472421010280sup002.docx]

**S2. Studies excluded at data extraction and results of author contact**

| **Author** | **Article Name** | **Contact author successful?** | **Author’s reply** |
| --- | --- | --- | --- |
| Alexopoulos et al. 2013 | Personalised intervention for people with depression and severe COPD | Yes | Study does not meet SDM criteria. |
| Aragones et al. 2009 | Implementing collaborative care for depression treatment in primary care: a cluster randomized evaluation of a quality improvement practice redesign | No |  |
| Dobscha et al. 2006 | Depression Decision Support in Primary Care: A Cluster Randomized Trial | Yes | Study does not meet SDM criteria. |
| Dunlop et al. 2012 | Depression beliefs, treatment preference, and outcomes in a randomized trial for major depressive disorder | Yes | Study does not meet SDM criteria. |
| Dwight-Johnson et al. 2010 | Effectiveness of collaborative care in addressing depression treatment preferences among low-income Latinos | No |  |
| Eli et al. 2008 | Randomized controlled trial of collaborative care management of depression among low-income patients with cancer | No |  |
| Eli et al. 2011 | One-year postcollaborative depression care trial outcomes among predominantly Hispanic diabetes safety net patients | No |  |
| Katon et al. 2015 | A randomized trial of collaborative depression care in obstetrics and gynecology clinics: socioeconomic disadvantage and treatment response | No |  |
| Kravitz et al. 2013 | Patient engagement programs for recognition and initial treatment of depression in primary care: a randomized trial | Yes | Study does not meet SDM criteria. |
| Lin et al. 2005 | The influence of patient preference on depression treatment in primary care | No | Study does not meet SDM criteria. |
| Melville et al. 2014 | Improving care for depression in obstetrics and gynecology: a randomized controlled trial | No | Study does not meet SDM criteria. |
| Pyne et al. 2010 | Cost-effectiveness analysis of a rural telemedicine collaborative care intervention for depression | No | Study does not meet SDM criteria. |
| Sharpe et al. 2014 | Integrated collaborative care for comorbid major depression in patients with cancer (SMaRT Oncology-2): a multicentre randomised controlled effectiveness trial | Yes | Study does not meet SDM criteria. |
| Stewart et al. 2014 | Effect of collaborative care for depression on risk of cardiovascular events: data from the IMPACT randomized controlled trial | No |  |
| Vergouwen et al. 2009 | Improving patients' beliefs about antidepressants in primary care: a cluster-randomized controlled trial of the effect of a depression care program | No |  |
| Vigod et al. 2019 | A patient decision aid for antidepressant use in pregnancy: Pilot randomized controlled trial | Yes | Study does not meet SDM criteria. |
