# Supplementary material for: The impact of shared decision-making on the treatment of anxiety and depressive disorders: systematic review
Source: BJPsych Open. 2021 Oct 7;7(6):e189. doi: 10.1192/bjo.2021.1028 (PMC8517854; doi:10.1192/bjo.2021.1028)
Supplement: Supplementary file 1 [file bjosup.zip › S2056472421010280sup003.docx]

**S1.** MEDLINE Search Strategy

1. *Decision Making/

2. Shared decision making.ti,ab.

3. decision support.ti,ab.

4. patient participation.ti,ab.

5. Patient Participation/

6. patient involvement.ti,ab.

7. *Patient Preference/

8. patient preference.ti,ab.

9. patient engagement.ti,ab.

10. patient perspective.ti,ab.

11. or/1-10

12. exp Depression/

13. exp Depressive Disorder/

14. Dysthymic Disorder/

15. depression.ti,ab.

16. exp Anxiety/

17. exp Anxiety Disorders/

18. (anxiety or phobia or phobic or panic).ti,ab.

19. or/12-18

20. 11 and 19

21. limit 20 to humans

22. limit 21 to english language
